# Implementation of genetic diagnosis and personalized management of hereditary angioedema in a Chinese regional center: a community case study of three families

**DOI:** 10.3389/falgy.2025.1696666

**Published:** 2025-11-12

**Authors:** Wenjin Du, Zhaoji Meng, Ke Yang, Qiuxing Zhang, Xianghua Lin, Wenchao Zhang, Weili Guo, Siqin Wang

**Affiliations:** 1 Department of Allergy, Henan Provincial People’s Hospital, Zhengzhou, China; 2 Department of Allergy, People’s Hospital of Zhengzhou University, Zhengzhou, China; 3 Department of Allergy, People’s Hospital of Henan University, Zhengzhou, China; 4 Medical Genetics Institute, Zhengzhou University People’s Hospital, Zhengzhou, China; 5 Henan Key Laboratory of Genetic Diseases and Functional Genomics, People’s Hospital of Henan University, Zhengzhou, China

**Keywords:** hereditary angioedema, c1 inhibitor, *SERPING1*, community case study, genetic diagnosis, China

## Abstract

**Background:**

Hereditary angioedema (HAE) remains significantly underdiagnosed and misdiagnosed in China, with laryngeal involvement leading to high mortality rates, creating an urgent need for exploring feasible diagnostic and management approaches in resource-limited settings.

**Objectives:**

To establish and evaluate a community-oriented comprehensive HAE diagnosis and management program at a regional center in central China; characterize the clinical and biochemical phenotypes of three unrelated families; identify *SERPING1* variants; implement personalized treatment and family cascade screening; and evaluate key program elements as a proof-of-concept model.

**Methods:**

From September 2022 to August 2025, we established a systematic workflow for suspected HAE cases at Henan Provincial People's Hospital, integrating clinical assessment, biochemical testing, and genetic analysis. Three unrelated families (45 subjects total) were enrolled. The program included standardized clinical assessment and real-time biochemical screening (C4, C1 inhibitor concentration/function), targeted *SERPING1* sequencing and variant classification American College of Medical Genetics and Genomics (ACMG), family cascade screening and genetic counseling, stratified personalized treatment (on-demand icatibant and lanadelumab prophylaxis), electronic follow-up Angioedema Control Test (AECT)/Angioedema Quality of Life Questionnaire (AE-QoL), and quality management [Standard Operating Procedures (SOPs) and provincial External Quality Assessment (EQA) planning)].

**Results:**

Family 1: A heterozygous missense variant c.1034G > A (p.Gly345Glu) was detected in exon 7 of *SERPING1* in the proband, absent in 13 unaffected family members. Laboratory tests showed decreased serum C4 and C1INH levels with reduced functional activity, consistent with HAE type 1. Family 2: Three affected members carried the same heterozygous missense variant c.1396C > T (p.Arg466Cys) in exon 8 of *SERPING1*, absent in 8 unaffected members. Despite elevated C1INH antigen levels, functional activity was markedly reduced, establishing HAE type 2 diagnosis. Family 3: Three affected members carried the same heterozygous missense variant c.1483G > A (p.Val495Ile) in exon 8 of *SERPING1*, absent in 17 unaffected members. Laboratory tests showed decreased serum C4 and C1INH levels with reduced functional activity, consistent with HAE type 1. Personalized treatment strategies achieved good disease control: mild cases with on-demand icatibant; severe phenotypes with lanadelumab prophylaxis. During 12–25 months of follow-up, the four symptomatic patients showed markedly reduced attack frequency with no life-threatening events.

**Discussion & conclusion:**

This community program represents a proof-of-concept demonstrating what is possible in establishing specialized HAE services in resource-limited settings. Key facilitating factors included cascade screening and genetic counseling, standardized testing pathways and variant classification, flexible prophylactic strategies adapted to economic conditions, and electronic quality and outcome monitoring. This program has expanded the domestic *SERPING1* variant spectrum and provides preliminary insights and references for the future development of HAE services.

## Introduction

Hereditary angioedema (HAE) is a rare, life-threatening autosomal dominant genetic disease characterized by recurrent, unpredictable episodes of angioedema, with an estimated population prevalence of approximately 1/50,000 (1), individuals without ethnic or gender predilection, HAE remains significantly underdiagnosed, though epidemiological data remains lacking in China.

The pathophysiology of HAE primarily involves dysregulation of the contact activation system due to deficiency or dysfunction of C1 inhibitor (C1INH), a serine protease inhibitor encoded by the *SERPING1* gene location (GRCh38) is 11:57,597,685–57,614,848 at 11q12.1 (2, 3). C1INH serves as the primary regulator of multiple proteolytic cascade systems, including the complement, contact, coagulation, and fibrinolytic pathways. Its deficiency leads to uncontrolled activation of the contact system, resulting in excessive bradykinin generation, increased vascular permeability, and subsequent angioedema formation (1, 4).

Based on biochemical phenotype, HAE with C1INH deficiency is classified into two principal subtypes. Type 1 HAE, accounting for approximately 85% of cases, is characterized by reduced C1INH antigen levels and functional activity. Type 2 HAE, comprising 15% of cases, presents with normal or elevated C1INH antigen levels but diminished functional activity (2, 3). A third category, HAE with normal C1INH (HAE-nC1INH), has been identified in patients with typical clinical manifestations but normal C1INH parameters and no detectable *SERPING1* variants, often associated with variants in other genes. In recent years, researchers have successively identified several new pathogenic genes associated with HAE, including the genes encoding coagulation factor XII (*F12*) (5–7), angiopoietin-1 (*ANGPT1*) (8), plasminogen (*PLG*) (9, 10), kininogen-1 heavy chain (*KNG1*) (11), myoferlin (*MYOF*) (12), heparan sulfate glucosamine 3-O-sulfotransferase 6 (*HS3ST6*) (13), carboxypeptidase N (*CPN*) (14), and DAB2 interacting protein (*DAB2IP*) (15). Additionally, some patients still have unidentified pathogenic genes (HAE-unknown, HAE-UNK).

Over 700 pathogenic *SERPING1* gene variants have been identified to date (16). The predominant variant types are missense variants, followed by frameshift variants, small insertions and deletions, large genomic rearrangements, splice site defects, nonsense variants, and regulatory variants (3). In the Chinese population, variants underlying HAE exhibit considerable diversity. Missense variants and in-frame deletions represent the most prevalent types, comprising 36.8% of all variants, followed by frameshift variants (28.9%), nonsense variants (14.5%), splice site variants (13.2%), and large insertions and deletions (6.6%) (17). The mutational spectrum exhibits considerable heterogeneity, with approximately 30%–34% of cases arising from *de novo* variants, presenting diagnostic challenges in patients without positive family history (17, 18).

In China, HAE research commenced in 1980, with subsequent studies revealing that virtually all confirmed cases are attributable to *SERPING1* variants. Chinese cohort studies indicate a predominance of type 1 HAE (98.73%) with type 2 representing only 1.27% of cases (19). Notably, the incidence of laryngeal edema among Chinese HAE patients reaches as high as 55.06%, with an average time from edema onset to asphyxia of 4.6 h, a maximum mortality rate of 40%, and a misdiagnosis rate of 75%. Untreated individuals exhibit marked disease progression between 20 and 29 years of age or succumb to sudden laryngeal edema at a median age of 46 years (17, 19, 20), underscoring the paramount importance of early diagnosis and appropriate management.

Recent advances in genetic testing methodologies have facilitated the identification of cryptic pathogenic variants, including deep intronic variants, non-canonical splicing variants, and dominant-negative missense variants that may be missed by conventional sequencing approaches (21, 22). These findings have expanded our understanding of HAE molecular pathogenesis and highlighted the importance of comprehensive genetic analysis in suspected cases.

This study presents a proof-of-concept model of HAE diagnosis and management in a resource-limited healthcare environment. Specifically, we established a comprehensive HAE diagnosis and management program at a regional medical center in central China, we typify the systemic diagnostic obstacles: patients experiencing persistent misdiagnoses, unnecessary surgical interventions, and substantial economic burdens associated with seeking specialized medical care. These real-world challenges underscore the urgent need for establishing a professional diagnostic and treatment system. To address these challenges, the project innovatively integrated a multidimensional diagnostic and therapeutic strategy: standardized clinical assessment, real-time biochemical screening (C4, C1INH concentration/function), and targeted *SERPING1* sequencing, embedding family cascade screening and genetic counseling. Our innovation rationale lies in integrating standardized testing, variant classification, cascade screening, personalized treatment, and electronic follow-up into an “affordable, implementable, and evaluable” continuous service within a resource-limited regional environment, demonstrating what is possible in addressing the core challenge of this community case study.

Notably, on May 11, 2018, the National Health Commission and five other departments jointly formulated the “First List of Rare Diseases,” which officially incorporated HAE, providing a critical policy foundation for the development of disease diagnosis and treatment systems and creating institutional support for our research work.

## Materials and methods

### Study setting

Henan Provincial People's Hospital Department of Allergic Diseases (Provincial Allergy and Immunological Disease Diagnosis, Treatment and Quality Control Center), serving a population >99 million. Before program initiation, our hospital lacked systematic HAE diagnostic testing, requiring suspected patients to seek care elsewhere, imposing significant economic and accessibility burdens. To address this situation, the hospital implemented multiple strategies: First, the hospital has independently developed serum C4 and C1INH concentration testing (approximately $7.4). Second, by participating in the China Primary Healthcare Foundation's “Prescription Care—Collaborative Screening—C1 Esterase Inhibitor Detection Project,” free serum C4 and C1INH concentration/functional testing is provided for eligible patients.

This study represents a proof-of-concept pilot project aimed at demonstrating what is possible in establishing HAE diagnostic and management services within resource-constrained healthcare environments, with the explicit understanding that the model is not immediately generalizable. The project specifically focused on workflow feasibility and family-based case ascertainment through referred kindreds and cascade screening, deliberately diverging from population-based epidemiological investigations. Despite the extensive administrative catchment area of the medical center, active screening was intentionally restricted in scope. Consequently, the limited number of confirmed cases primarily reflects the region's known systemic diagnostic underestimation and referral constraints, rather than representing the true epidemiological prevalence of HAE.

The study protocol was approved by the Henan Provincial People's Hospital Institutional Ethics Committee (Approval No.: 2022-2211273107). Written informed consent was obtained from adults; parental consent and age-appropriate assent were obtained for minors.

Study subjects: From September 2022 to August 2025, three unrelated families totaling 45 individuals were enrolled, with complete three-generation pedigrees constructed.

### Diagnostic criteria

HAE diagnosis was established according to the Chinese Expert Consensus on Diagnosis and Treatment of Hereditary Angioedema (2020) and the International WAO/EAACI Guideline for Hereditary Angioedema Management—2021 Revision and Update (4, 23).

### Workflow and diagnostic pathway

Standardized clinical assessment: Structured history (age of onset, attack frequency/location/triggers, previous misdiagnoses and treatments, family history). Physical examination performed during both symptomatic and asymptomatic periods; attack-phase imaging obtained when feasible.

Serum C4, C1INH concentration and function determination: C4 and C1INH concentrations quantified by nephelometry; C1INH functional activity measured by ELISA. Peripheral venous blood (2 mL) collected from probands and all family members in three families for these tests.

DNA extraction: Peripheral venous blood (2 mL) collected from probands and all family members in three families. Genomic DNA extracted according to kit instructions (DNeasy Blood & Tissue Kit, Qiagen, Germany, Cat. No. 69504).

PCR Amplification: Primers were designed using the online software Primer3 (https://bioinfo.ut.ee/primer3-0.4.0/) to amplify the promoter, exon, and intron regions of the *SERPING1* gene. Primer sequences and amplicon lengths shown in Table 1 (all reactions 35 cycles).

**Table 1:** Primer sequences and PCR conditions.

Region	Primer Sequence (5′→3′)	PCR product length (bp)	Ta Opt (℃)
5UTR	GGCCAGCCAATAGCTAAGAC	246	58
	AGTCCCAGGTGGAAGCAAG		
Exon1	GAGGAGCCAGGGAGAAGGT	391	58
	ACCATCTACGAGTGCGTGTG		
Intron1	ACTCTCTACTCAGTCTGCACT	658	60
	GACCACATACCCCAGCCAG		
Exon2	CCACACCTTCTCTTCCTGCT	599	59
	CTTCTTCCCAGAGGCATGG		
Intron2	CTGACCCTGCTGACCCTC	1,814	58
	GGTTGTGTGGTGGGTTCATC		
Exon3	GATCTGTGATCCCCTCCAAA	360	58
	GCTCCAACATTCCCTCTGTC		
Intron3	CTTCTGCCCAGGACCTGTTA	1,986	60
	TGGGCTGTGGAAGATCTGAG		
Exon4	CCACCATGCCGTATTCACTA	491	59
	TGCAGGTAGGAAAAGGATGAA		
Intron4-1	ACTTCACCTGTGTCCACCAG	1,938	58
	AGGCGCGTGCCACCACACCTG		
Intron4-2	GTGACAGACCGAGACTCTGTCTC	1,976	58
	TGTTGCTTAGGACTCTGGGG		
Exon5	AGCCCACTTAACCCCAAGTT	356	58
	CCCCAAAATGATGGGACTAC		
Intron5	GACAGTCTGCCCTCCGATAC	290	60
	CATTCTGGTTTTCTTGGGATCAA		
Exon6	CAGGAGAGAGATGCGGTAGG	396	58
	AGGGTTGCAGGACAAACTGA		
Intron6-1	TGTGGCCCATTTCATTGACC	1,392	58
	AGATGGGTGGATAAGTCCAT		
Intron6-2	GCCCGCCACCACACCTGGCT	2,117	58
	TTCAACATCATGTTAAAACC		
Intron6-3	CATCTAATACCTGACAGGTC	1,898	58
	TCACTTTGATGCGGGGTAGT		
Exon7	AGAGAGCACTGGGACTCAGG	376	60
	GGAGCCCTTTTGGTGGATAG		
Intron7	ACTACCCCGCATCAAAGTGA	2,563	59
	CACCCCAGTCTCTGTCAGTT		
Exon8 + 3UTR	AGCAGCACAAGTTCCCTGTC	399	58
	AGCCTGGGTGACAGATTGAG		

Sanger Sequencing: PCR products were purified, and bidirectional sequencing was performed on the purified amplification products using an ABI-3500DX sequencer (Applied Biosystems, USA).

Bioinformatics Analysis: REVEL (https://sites.google.com/site/revelgenomics/) was used to predict variant pathogenicity. Variants were interpreted according to the 2015 ACMG Standards and Guidelines for the Interpretation of Sequence Variants (24). Evidence strength categories were weighted as very strong (PVS), strong (PS), moderate (PM), and supporting (PP).

**Figure 1:**
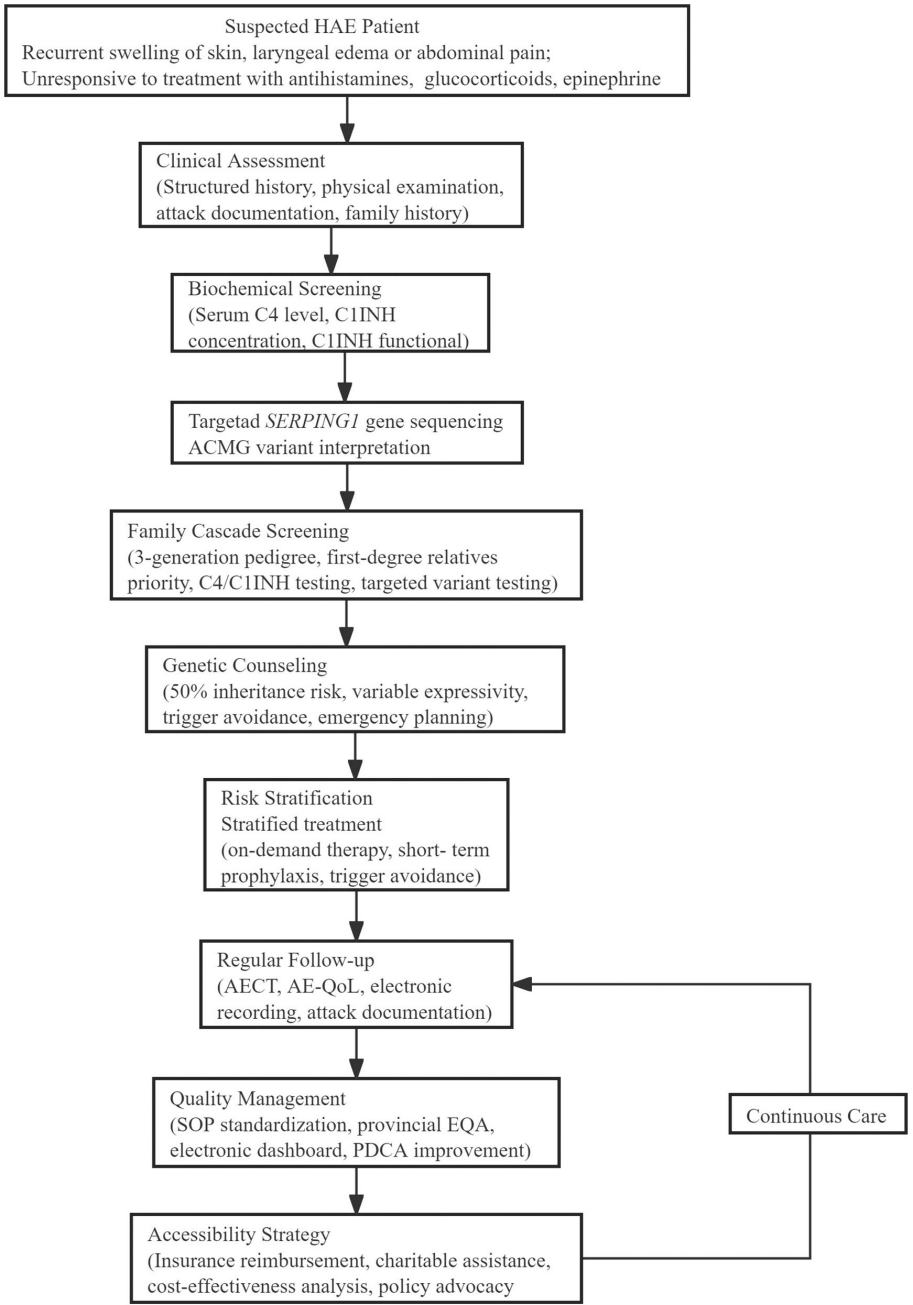
Study flow chart.

Detailed diagnostic and therapeutic flowchart (Figure 1).

## Materials and methods—genetic testing and segregation

Cascade screening strategy: Construct a pedigree spanning at least three generations; for co-segregation analysis, include only biological relatives. Spouses and other non–blood-related individuals may be tested for counseling purposes but are excluded from co-segregation counts. Prioritize first-degree relatives; with informed consent, perform C4 and C1INH concentration/function assays, and conduct targeted genetic testing for the family-specific variant established in the proband.

Counseling points: 50% autosomal dominant inheritance risk, variable expressivity and age-related expression, reproductive options, trigger avoidance and prodrome recognition, personalized emergency plans, and compliant communication with written notification of potential insurance and employment implications.

### Stratified personalized treatment and local adaptation

Mild/low burden: On-demand treatment (icatibant 30 mg subcutaneous), with pre-filled syringes and family emergency plans provided.

Severe/high risk: Those with laryngeal or gastrointestinal involvement prioritized for long-term prophylaxis (lanadelumab 300 mg subcutaneous every 2 weeks; extended to every 4 weeks under economic constraints with close AECT monitoring and breakthrough treatment assurance).

Monitoring tools: Regular assessment with AECT (disease control) and AE-QoL (quality of life); electronic recording of attacks and medications.

## Quality and data systems

SOP standardization: End-to-end SOP and version management from blood collection/transport, testing and reporting, ACMG interpretation to follow-up and medication pathways.

Provincial EQA (planning): Regular proficiency testing and closed-loop improvement to enhance inter-institutional result comparability.

Electronic follow-up and dashboard: Structured capture of attack rates, laryngeal events, emergency/hospitalization, medications and adverse events; key quality indicators [Turnaround Time (TAT), EQA scores, AECT completion rate/scores, emergency rates, prophylaxis adherence, etc.] visualized, supporting Plan–Do–Check–Act (PDCA) continuous improvement.

### Accessibility and payment strategies

Payment and assistance: Through the China Primary Health Care Foundation, we implemented an innovative co-payment model integrating medical insurance and foundation assistance, strategically designed to mitigate out-of-pocket expenses for long-term prophylaxis and emergency medications in HAE treatment. This financial intervention significantly reduced patients’ economic burden, successfully limiting patient self-payment proportions to 10%–30% for critical treatments, with medication costs specifically reduced to approximately $207 per dose for Lanadelumab and $194 per dose for Icatibant. Economic evaluation: Conducting cost-effectiveness and equity evaluation of stratified prevention strategies (e.g., cost per laryngeal event avoided, payment burden by income stratification) to provide evidence for policy decisions.

## Results

### Clinical characteristics of families

**Figure 2:**
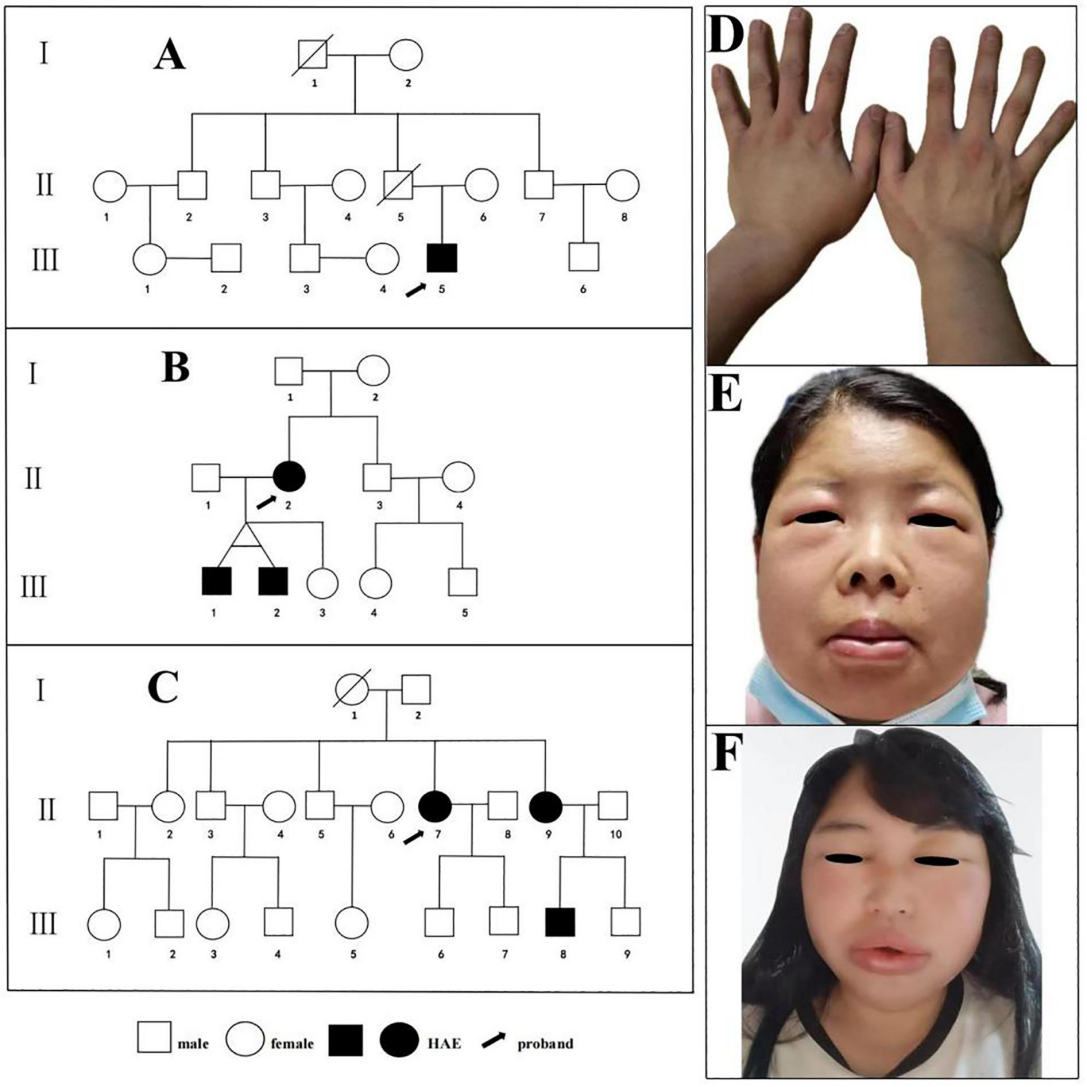
Pedigrees of the three families with HAE. **(A)** Hereditary Angioedema Family 1. **(B)** Hereditary Angioedema Family 2. **(C)** Hereditary Angioedema Family 3. Images of acute attacks in index patients from three Hereditary Angioedema families. **(D)** The proband of Family 1 presenting with left hand edema; **(E)** The proband of Family 2 presenting with edema of the lips, eyelids, and face. **(F)** The proband of Family 3 presenting with edema of the lips, eyelids, and face.

Family 1 (*n* = 14): Three-generation family with 14 members evaluated (Figure 2A). Proband (III-5), male, 24 years old, presented with recurrent non-pitting edema predominantly affecting upper limbs for 4 years, occurring approximately 3–5 times annually, self-resolving without treatment over 1–3 days. Triggers included physical activity, mechanical pressure, and minor trauma. Previously misdiagnosed with chronic urticaria, unresponsive to antihistamines. Presented with left hand non-pitting edema one day before enrollment (Figure 2D). Family history: grandfather (I-1) died of laryngeal edema asphyxiation at age 63; uncle (II-7) had similar but less frequent attacks; father (II-5) died of hepatocellular carcinoma without clear edema history.

Family 2 (*n* = 11): Three-generation family with 11 members evaluated (Figure 2B). Proband (II-3), female, 36 years old, experienced recurrent facial, limb, and gastrointestinal edema for 20 years. Attacks occurred 8–10 times annually, with facial and limb edema lasting 3–5 days; abdominal attacks presented as severe cramping, nausea, and vomiting lasting 2–3 days. Previously underwent exploratory laparotomy for suspected acute abdomen and multiple hospitalizations for misdiagnosed acute pancreatitis or appendicitis. Presented with lip, eyelid, and facial edema 2 days before enrollment (Figure 2E). Her twin sons (III-1, III-2, 9 years old) were asymptomatic at evaluation but showed biochemical abnormalities consistent with HAE.

Family 3 (*n* = 20): Three-generation family with 20 members evaluated (Figure 2C). Proband (II-7), female, 43 years old, experienced recurrent facial and limb edema with occasional laryngeal edema for 4 years. Attacks occurred 7–10 times annually, with facial and limb edema lasting 3–5 days; underwent tracheal intubation for laryngeal edema 2 years ago, with multiple hospitalizations for misdiagnosed acute laryngitis and edema. Presented with lip, eyelid, and facial edema 10 h before enrollment (Figure 2F). Her sister (III-9, 37 years old), has a 5-year history of recurrent extremity edema occasionally accompanied by abdominal pain, with approximately 5–6 attacks per year, no apparent triggers, and episodes resolving spontaneously in about 2 days without treatment. Her nephew (IV-8, 11 years old), was asymptomatic at evaluation but had biochemical abnormalities consistent with HAE.

Previous medical history, clinical symptoms, treatment regimens, and edema control status of the four patients with HAE are shown in Table 2.

**Table 2:** Clinical characteristics of symptomatic patients with HAE.

Clinical Data		Subject			
		Proband 1	Proband 2	Proband 3	Proband 3's younger sister
Sex		Male	Female	Female	Female
Age(years)		26	38	43	37
HAE type		HAE-1	HAE-2	HAE-1	HAE-1
Family history		None	Positive	Positive	Positive
Age at first onset (years)		22	16	39	32
Time from onset to diagnosis (years)		2	20	4	5
Edema attack sites		Limbs	Face, limbs, abdomen, pharynx	Face, larynx, limbs	Abdomen, limbs
Triggering factors		Exertion, compression, minor trauma	Cold exposure, emotional changes	Without apparent trigger	Without apparent trigger
Duration (days)		1–2	2–5	3–5	2–3
Attacks/year	Base-line	5	14	13	9
	Post-treatment	2	1	0	0
AECT scores	Base-line	11	4	2	5
	Post-treatment	12	13	13	14
Edema severity during attacks		Mild	Severe (requiring bed rest)	Severe (requiring bed rest)	Severe
Urticaria during attacks (Yes/No)		No	No	No	No
Current treatment (Danazol/Icatibant/Lanadelumab)		Icatibant for on-demand/acute attack treatment (30 mg per attack/dose)	(1) 2022.09.29-2023.07.30: Danazol 200 mg twice daily (oral), taken inconsistently. (2) 2023.11.20- present: lanadelumab 300 mg subcutaneously every 4 weeks (q4w).	(1) 2024.09.03-2024.12.10: lanadelumab 300 mg subcutaneously every 2 weeks (q2w). (2) 2024.12.27-present: lanadelumab 300 mg subcutaneously every 4 weeks (q4w).	(1) 2024.09.21-2024.12.19: lanadelumab 300 mg subcutaneously every 2 weeks (q2w). (2) 2025.01.07-present: lanadelumab 300 mg subcutaneously every 4 weeks (q4w).

### Biochemical parameters

Family 1: Proband's serum C4 and C1INH functional activity were significantly below 50% of normal lower limits, consistent with HAE type 1 diagnosis. Test results for 13 normal controls within the family were all within reference ranges.

Family 2: Proband and her two sons (III-1, III-2, both asymptomatic) showed elevated serum C1INH antigen levels with reduced functional activity, consistent with HAE type 2 diagnosis. Test results for 8 normal controls within the family were all within reference ranges.

Family 3: Proband, her sister, and nephew showed serum C4 and C1INH functional activity significantly below 50% of normal lower limits, consistent with HAE type 1 diagnosis. Test results for 17 normal controls within the family were all within reference ranges.

C4 and C1INH level of serum and C1INH function, for the seven HAE patients across the three families are presented in Table 3.

**Table 3:** Results of the C4 and C1INH level of serum and C1INH function in patients with HAE.

Subject		Laboratory test (normal rages)		
		C4 level (g/L)	C1INH level (g/L)	C1INH function（%）
Family 1	Proband 1	0.03	0.11	2.5
Family 2	Proband 2	0.02	0.7	6.3
	Proband 2's elder son	0.08	0.59	60.8
	Proband 2's younger son	0.10	0.62	51.6
Family 3	Proband 3	0.02	0.08	13.1
	Proband 3's younger sister	0.04	0.06	19.7
	Proband 3's nephew	0.06	0.19	53.4

The normal range for C4 levels is 0.10–0.40 g/L, C1INH levels is 0.21–0.39 g/L, and normal C1INH function is defined as equal to or greater than 68%.

### Genetic analysis

#### *SERPING1* variant identification

**Figure 3:**
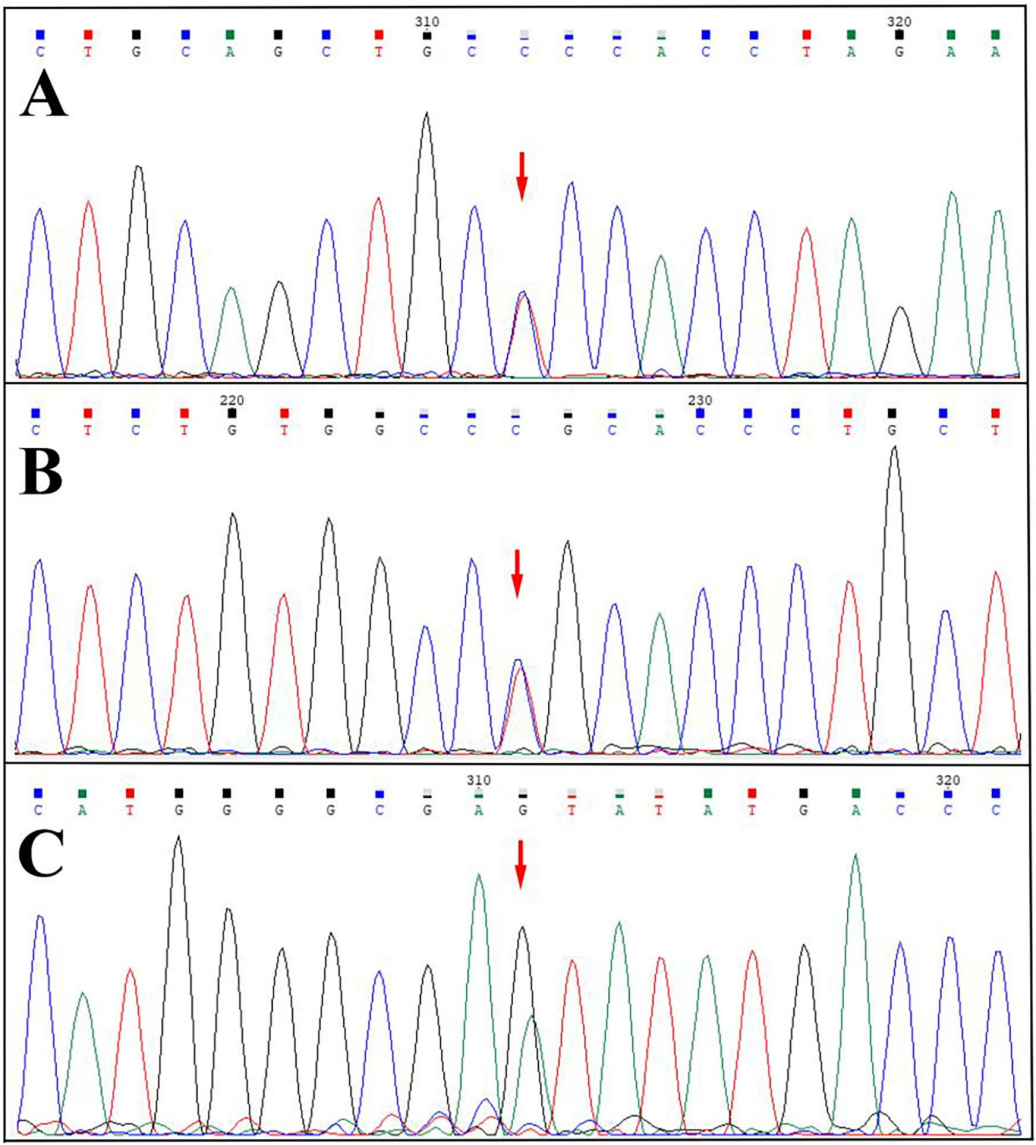
Sequencing map. **(A)** Heterozygous missense variant of the *SERPING1* gene (NM_000062.3) c.1034G>A (p.Gly345Glu) in the proband 1 (reverse sequencing). **(B)** Heterozygous missense variant of the *SERPING1* gene (NM_000062.3) c.1396C>T (p.Arg466Cys) in the proband 2. **(C)** Heterozygous missense variant (NM_000062.3) c.1483G > A (p.Val495Ile) in the proband 3.

*SERPING1* gene testing results for probands and confirmed HAE patients in three families (Figure 3).

Family 1 (Figure 3A): Proband carried heterozygous missense variant (NM_000062.3) c.1034G>A (p.Gly345Glu) in *SERPING1* exon 7, causing glycine to glutamic acid substitution at position 345. All 13 normal controls within the family were wild-type, without this variant.

Family 2 (Figure 3B): Proband carried heterozygous missense variant (NM_000062.3) c.1396C>T (p.Arg466Cys) in *SERPING1* exon 8, causing arginine to cysteine substitution at position 466. Her two sons (III-1, III-2) also carried this heterozygous variant. All 8 normal controls within the family were wild-type, without this variant.

Family 3 (Figure 3C): Proband carried heterozygous missense variant (NM_000062.3) c.1483G > A (p.Val495Ile) in *SERPING1* exon 8, causing valine to isoleucine acid substitution at position 495. Her sister and nephew (III-9, IV-8) also carried this heterozygous variant. All 17 normal controls within the family were wild-type at this locus, without this variant.

### Bioinformatics analysis and variant classification

Family 1: *SERPING1* c.1034G > A. Annotated as “Pathogenic” in ClinVar (Variation ID: 2137099). No carrier frequency observed in East Asian populations in gnomAD_exome database (PM2_Supporting). Multiple pathogenic variants reported near this locus (PM1_Supporting) (25, 26). This variant detected in an unrelated HAE patient (PS4_Supporting) (27). REVEL prediction score 0.794, meeting supportive pathogenic evidence (PP3_Moderate). The proband exhibits typical C1INH deficiency and reduced functional activity, accompanied by a characteristic clinical phenotyp (PP4_Supporting). According to ACMG variant classification standards, this variant classified as “Likely Pathogenic” (PM2_Supporting + PP3_Moderate + PS4_Supporting + PM1_Supporting + PP4_Supporting).

Family 2: *SERPING1* c.1396C > T. Variant occurs in functional domain (PM1_Moderate). Annotated as “Pathogenic” in ClinVar (Variation ID: 3947) and “Disease-causing variant” in HGMD. No carrier frequency in East Asian populations in gnomAD_exome (PM2_Supporting). Variant detected in multiple unrelated HAE patients (PS4_Strong) (17, 28–31). REVEL score 0.721, meeting supportive pathogenic evidence (PP3_Supporting). The proband had elevated C1INH antigen levels with markedly reduced functional activity and presented with severe, HAE-specific clinical manifestations, including facial angioedema and colicky abdominal pain (PP4_Supporting). According to ACMG standards, this variant classified as “Pathogenic” (PM1_Moderate + PM2_Supporting + PP3_Supporting + PS4_Strong + PP1_Supporting + PP4_Supporting).

Family 3: *SERPING1* c.1483G > A not recorded in ClinVar, HGMD, or LOVD, with no carrier frequency in East Asian populations in gnomAD_exome database (PM2_Supporting). The variant perfectly co-segregated with HAE in a three-generation family (PP1_Supporting). The proband presented with severe, HAE-specific manifestations including laryngeal edema (PP4_Supporting). However, computational prediction yielded a low REVEL score of 0.12, suggesting benign impact (BP4_Supporting). According to ACMG standards, this variant classified as “Variant of Uncertain Significance” (PP1_Supporting + PM2_Supporting + PP4_Supporting + BP4_Supporting).

### Local adaptation of treatment protocols

#### Personalized management strategies

Treatment selection comprehensively considered disease severity, attack frequency, laryngeal involvement history, and economic factors:

Mild phenotype (Family 1): Acute attacks treated on-demand with icatibant 30 mg subcutaneous. Patient trigger avoidance education with pre-filled syringes and emergency action plans.

Severe phenotype (Family 2): Previous laryngeal and gastrointestinal involvement, initiated lanadelumab long-term prophylaxis (300 mg subcutaneous every 4 weeks due to economic reasons; standard interval every 2 weeks). Breakthrough therapy: icatibant availability; ongoing monitoring with the AECT and the AE-QoL questionnaire.

Severe phenotype (Family 3): Previous laryngeal and facial involvement, initiated lanadelumab long-term prophylaxis (300 mg subcutaneous every 2 weeks, adjusted to every 4 weeks after 6 months). Breakthrough therapy: icatibant availability; AECT. Her sister, with prior extremity and gastrointestinal involvement, likewise initiated long-term prophylaxis with the same lanadelumab regimen, with availability of icatibant for breakthrough attacks and ongoing monitoring using the AECT and the AE-QoL questionnaire.

#### Follow-up and outcome monitoring

Family 1: During 25 months follow-up, proband experienced 4 mild limb edema episodes, all successfully controlled with icatibant, with symptom resolution within 2–4 h post-administration.

Family 2: During 21 months follow-up, proband experienced only 1 buttock edema episode, possibly related to prolonged sitting, resolving within approximately 2 h. At each visit over the past 6 months, AECT scores were ≥13, and the 4-week AE-QoL score was <11, indicating good control. Icatibant available for breakthrough attacks. Two asymptomatic sons received no prophylactic treatment but comprehensive trigger avoidance and early symptom recognition education with continued observation.

Family 3: During 12 months of follow-up, the proband had no angioedema attacks; at each visit over the past 6 months, AECT scores were ≥13, and the 4-week AE-QoLscore was <6, indicating good control, and icatibant was available for breakthrough attacks. Her sister likewise had no angioedema attacks during 12 months of follow-up (AECT scores at each visit were ≥14, and the 4-week AE-QoL score was <3). Her nephew was asymptomatic and not on prophylactic therapy; alongside disease education and follow-up management, he was provided with on-demand emergency medication (icatibant) for immediate access.

## Discussion

This community case study established an integrated service pathway combining “standardized biochemistry—targeted genetics—cascade screening—personalized treatment—electronic follow-up—quality management” at a regional center hub, demonstrating the potential feasibility of establishing specialized HAE services in resource-limited settings within a pilot framework. Evaluation of three unrelated families revealed HAE's phenotypic heterogeneity and age-related variability, demonstrating that family-directed testing can enhance early identification and enable risk-based management.

Our findings highlight major diagnostic gaps in resource-limited settings—including frequent misdiagnoses, unnecessary surgeries, and high economic burden—underscoring the need for standardized diagnostic and management frameworks. Since March 2023, our center has participated in the national HAE Diagnostic Capacity Standardization Project, aiming to improve clinical outcomes and quality of life. The project remains exploratory, requiring additional resource and policy support for broader implementation. In developing countries, genetic testing cost and accessibility pose specific challenges. Our approach establishes a pragmatic diagnostic pathway that prioritizes clinical phenotypes and biochemical markers (C4, C1INH concentration/function) as the first-line diagnostic basis, with genetic testing reserved for risk stratification and long-term management. This stepwise model balances cost, accessibility, and clinical accuracy, enabling feasible and equitable HAE diagnosis and treatment in constrained health systems. The clinical presentations in our cohort exemplified HAE phenotypic heterogeneity. The three probands’ earliest onset was at age 16, with the longest interval from onset to diagnosis reaching 20 years, consistent with domestic reports: average onset age 21.25 years, approximately 75% experiencing first attacks at ages 10–30, average diagnostic delay approximately 12.64 years (19). Delays reflect HAE's rarity and variable presentations, easily confused with common diseases. Due to low HAE awareness among physicians and patients, patients often seek multiple consultations without diagnosis. Initial presentations may involve airways or abdomen, risking asphyxiation or unnecessary surgical intervention. International studies show HAE patients average 4.4 physician consultations before diagnosis, 65% experience misdiagnosis, and 24% undergo unnecessary exploratory abdominal surgery (32). In Family 2, the proband underwent exploratory laparotomy for suspected acute abdomen and multiple hospitalizations for misdiagnosed acute pancreatitis/appendicitis. In Family 3, the proband underwent tracheal intubation for laryngeal edema. Many undiagnosed patients face higher mortality risk due to lack of timely diagnosis and treatment (33). China currently lacks data on emergency department HAE acute attack presentations.

Using three-generation pedigrees to identify high-risk relatives, implementing biochemical-genetic targeted testing for at-risk members, and embedding counseling into outpatient workflows significantly improved adherence and completion rates. We found two genetically confirmed children in Family 2 (III-1, III-2, twins, 9 years old) and one genetically confirmed patients in Family 3 (IV-8, 11 years old) had biochemical abnormalities but were clinically asymptomatic, demonstrating HAE's variable expressivity and age-related expression. HAE's clinical spectrum is extremely broad, from completely asymptomatic to life-threatening attacks; attack frequency and severity can differ significantly within the same family (19). Onset age varies greatly, with over 75% experiencing first attacks before age 30, typically worsening during ages 20–29 (19, 34). Previous studies suggest up to 30%–34% are *de novo* pathogenic variants, possibly without positive family history (17, 18, 35), thus negative family history does not exclude HAE. This supports early, parallel gene–biochemistry testing for at-risk relatives regardless of current symptom status. Once a familial pathogenic variant is defined, targeted Sanger sequencing provides a cost-effective cascade testing method, reducing turnaround time and unnecessary next-generation sequencing expenses. Integrating biochemical results and genetic confirmation allows same-visit triage, rapid decision-making, and implementation at regional laboratories with minimal infrastructure. From a public health standpoint, this family-directed dual-track strategy enhances early detection and cost-effectiveness in resource-limited settings. In our pilot, three asymptomatic carriers were identified prospectively, enabling risk stratification and preventive education. Beyond diagnostic yield, this strategy strengthens long-term management through anticipatory guidance and emergency readiness, reducing risk of severe attacks and emergency burden. Importantly, it complements rather than replaces comprehensive genetic testing, providing a phased and scalable framework adaptable to clinical and economic realities.

Treatment response differences among the three probands emphasized the importance of personalized treatment. In this study, Family 1's proband achieved success with on-demand icatibant for mild attacks, while Family 2 and 3 probands required long-term lanadelumab prophylaxis due to severe phenotypes. This aligns with current international guidelines advocating personalized treatment based on disease burden, attack frequency, and quality of life impact (4). Extending lanadelumab dosing intervals to every 4 weeks (standard every 2 weeks) due to economic considerations while still achieving adequate disease control highlights treatment accessibility issues in resource-limited settings. This underscores the critical issue of treatment access in resource-limited settings: with appropriate risk communication and close monitoring, dose-interval extension may serve as a context-specific trade-off under resource constraints. Although Non-standard dosing was effective in these cases; however, expanding access to modern HAE treatments remains urgent.

Management of HAE should be phenotype driven, implementing guideline-concordant on-demand therapy and indicated prophylaxis; a VUS should not serve as the sole basis for clinical decision making, and current molecular findings should be integrated into the evidence base with periodic reinterpretation. For patients with strong clinical and biochemical evidence of HAE but negative conventional sequencing, we recognize the importance of additional genetic analyses such as MLPA to detect large deletions/duplications; our future standard protocol will incorporate these comprehensive methods to identify all possible pathogenic mechanisms, ensuring complete molecular characterization while maintaining phenotype-based management decisions. Identifying pathogenic variants in asymptomatic carriers presents genetic counseling challenges. Among Families 2 and 3, three asymptomatic individuals carrying pathogenic variants should be enrolled in longitudinal, family-centered follow-up. In addition to systematic education on trigger avoidance, prodrome recognition, and emergency management, psychosocial support should be emphasized, including communication around uncertainty and anxiety with stress-reduction strategies, individualized guidance on academic and sports participation, facilitation of effective intra-family communication and caregiver stress management, and a readily actionable, always-available written emergency plan to mitigate fear and helplessness. For minors and their families, we will refine counseling points (trigger avoidance, prodrome recognition, emergency planning, and psychological coping) and specify the follow-up cadence: reassessment every 6–12 months during stable periods; every 3–6 months during puberty or other major physiologic/environmental transitions, prior to elective surgery or dental procedures, and at initiation or discontinuation of estrogen-containing therapies or angiotensin-converting enzyme inhibitors; with immediate evaluation for any prodromal symptoms or suspected attacks. Follow-up will include baseline profiling (definition of the familial subtype and individual risk; documentation of family history of attacks, potential trigger exposures, past medical and medication history), psychosocial screening (anxiety/depression, school and peer interactions, family stress), and health literacy assessment; laboratory monitoring will be tailored to subtype, with clinical surveillance prioritized in normal–C1INH forms and testing frequency adjusted pragmatically. At each visit, we will review symptom/trigger diaries, quality-of-life measures, and participation in learning and sports, and update the individualized emergency plan. Patient education and support systems will be strengthened through standardized materials and family meetings, training in stress-management and coping skills, and referral to psychological counseling or peer support as needed; we will also liaise with schools or childcare institutions, providing physician letters and emergency cards to reduce absenteeism and stigma and to optimize participation in school activities. Ongoing trigger avoidance and medication review will be maintained, with personalized travel and exercise advice and perioperative planning; access to and training in the use of on-demand medications will be ensured, medical alert identification provided, red-flag symptoms and care-seeking thresholds defined, and emergency contact and follow-up pathways established (including telehealth or rapid-access appointments). Finally, we will plan a smooth transition from pediatric to adult care and provide counseling on contraception and pregnancy management, favoring non-estrogen options and individualized care during pregnancy.

Notably, our findings expand the Chinese *SERPING1* pathogenic variant spectrum to 98 variants (17, 36–40), with c.1034G > A and c.1483G > A identified in Chinese patients for the first time, suggesting the domestic variant spectrum is broader than previously recognized. Our program also represents one of the first coordinated HAE service model in China. This further emphasizes that targeted locus testing within families can significantly reduce costs and turnaround time, suitable for large-scale cascade screening in resource-limited scenarios.

### Key program elements demonstrated in this pilot and considerations for pathway optimization

This proof-of-concept program successfully implemented several integrated elements under specific enablers—intensive case management, foundation-subsidized drug access, and strong institutional support. These measures demonstrate feasibility within a controlled pilot environment rather than universal applicability. Scaling up will require context-tailored adaptation, resource assessment, and capacity building aligned with local healthcare and economic realities.

Prioritizing C4 and C1INH function testing in suspected cases and family screening as low-cost, highly accessible “triage tools,” followed by family-directed sequencing, improves efficiency and affordability. Based on attack frequency, involved sites (especially laryngeal/gastrointestinal), AECT, AE-QoL and previous crisis history, adopting a stepwise strategy of “on-demand icatibant + breakthrough treatment assurance + lanadelumab prophylaxis”; under economic constraints, cautiously extending lanadelumab dosing intervals with close monitoring. Setting electronic medical record (EMR) embedded alerts for patients with “recurrent non-pitting edema,” “abdominal cramps with vomiting but inconsistent imaging/enzymes,” or “emergency laryngeal event history” triggers HAE screening workflows, reducing unnecessary exploratory surgery and intubation risks. Implement multidisciplinary, tiered care with coordinated primary-level referral pathways and teleconsultation to minimize repeat visits and avoidable interventions. A digital follow-up program should integrate attack events, emergency utilization, and medication responses into a visual dashboard to enable bidirectional alerts and early intervention. Scale-up should concurrently advance workforce training and infrastructure, drug-access strategies, and practical implementation at the awareness/cultural level, with concrete elements including SOP templates, training modules for primary hospitals, telemedicine support, EMR flags, and participation in EQA to ensure testing consistency. Address access barriers to icatibant and lanadelumab; through medical insurance coverage and foundation assistance, we significantly reduced patient economic burdens: the out-of-pocket cost for icatibant is approximately $194 (originally $607), and the out-of-pocket cost of lanadelumab is approximately $207 (originally $1,989).

## Limitations and risk control

Sample size and representativeness: Only 3 families (with 7 confirmed patients) were enrolled; referral-based sampling may overestimate severe phenotype proportions, limiting generalizability.

Follow-up duration: 12–25 months insufficient to assess long-term outcomes and complications; continued tracking needed to verify sustainability and safety.

Dosing strategy bias: Some individuals adopted non-standard interval prophylactic regimens; while effective under monitoring, cannot replace evidence-based standards; requires validation in larger samples with longer follow-up.

Finally, as a pre–post observational implementation study, this work is subject to inherent limitations related to small sample size, potential severity-related referral bias, and constraints on causal inference.

## Conclusion and future directions

This community case study demonstrates successful implementation of a comprehensive HAE diagnosis and management program at a Chinese regional center. Through systematic family screening, genetic characterization, and localized treatment protocols, we improved clinical outcomes and, for the first time in Chinese patients, identified two novel *SERPING1* variants.The program illustrates what is achievable in HAE service development within a resource-limited setting when supported by dedicated institutional commitment, subsidized medication access, and intensive case management. Our experience emphasizes the importance of local adaptation of international guidelines and provides insights into the opportunities and challenges of implementing specialized rare disease services in similar contexts. Notably, the gap between the administrative catchment population and the number of confirmed cases reflects underdiagnosis and early-phase program constraints rather than true prevalence. Continued expansion and optimization of such programs is needed to address the significant disease burden caused by HAE underdiagnosis and inadequate treatment in China.

### Future research directions

Multicenter prospective cohorts: Collaborating with inter-provincial centers to unify quantified outcomes (attack rates, laryngeal events, AECT/AE-QoL, emergency/hospitalization, and resource utilization) to generate real-world evidence.

Standardization and quality systems: Establishing provincial EQA, unified SOPs, digital follow-up systems, and quality indicator dashboards to make testing and treatment “more consistent, comparable, and sustainable.”

Accessibility and equity: Linking with insurance/charitable programs to systematically evaluate cost-effectiveness and equity of stratified prevention strategies, expanding modern HAE treatment accessibility through policy and payment innovations.

Progress and plans: Multicenter expansion and a prospective registry are underway; the multicenter cohort framework, harmonized outcome set, and registry timeline have been defined to further enhance external validity and dissemination/implementation value.

## Data Availability

The datasets generated for this study have been deposited in the ClinVar database under accession numbers Variation ID: 2137099 and Variation ID: 3947. These data are publicly available through the ClinVar repository at https://www.ncbi.nlm.nih.gov/clinvar/.

## References

[1] Busse PJ Christiansen SC. Hereditary angioedema. N Engl J Med. (2020) 382(12):1136–48. 10.1056/NEJMra1808012 32187470

[2] Santacroce R D'Andrea G Maffione AB Margaglione M d'Apolito M. The genetics of hereditary angioedema: a review. J Clin Med. (2021) 10(9):2023. 10.3390/jcm10092023 34065094 PMC8125999

[3] Sharma J Jindal AK Banday AZ Kaur A Rawat A Singh S Pathophysiology of hereditary angioedema (HAE) beyond the SERPING1 gene. Clin Rev Allergy Immunol. (2021) 60(3):305–15. 10.1007/s12016-021-08835-8 33442779

[4] Maurer M Magerl M Betschel S Aberer W Ansotegui IJ Aygören-Pürsün E The international WAO/EAACI guideline for the management of hereditary angioedema-the 2021 revision and update. Allergy. (2022) 77(7):1961–90. 10.1111/all.15214 35006617

[5] Bork K Wulff K Witzke G Hardt J. Hereditary angioedema with normal C1-INH with versus without specific F12 gene mutations. Allergy. (2015) 70(8):1004–12. 10.1111/all.12648 25952149

[6] Bork K Wulff K Witzke G Hardt J. Treatment for hereditary angioedema with normal C1-INH and specific mutations in the F12 gene (HAE-FXII). Allergy. (2017) 72(2):320–4. 10.1111/all.13076 27905115

[7] Prieto A Tornero P Rubio M Fernández-Cruz E Rodriguez-Sainz C. Missense mutation Thr309Lys in the coagulation factor XII gene in a Spanish family with hereditary angioedema type III. Allergy. (2009) 64(2):284–6. 10.1111/j.1398-9995.2008.01764.x 19178407

[8] Bafunno V Firinu D D'Apolito M Cordisco G Loffredo S Leccese A Mutation of the angiopoietin-1 gene (ANGPT1) associates with a new type of hereditary angioedema. J Allergy Clin Immunol. (2018) 141(3):1009–17. 10.1016/j.jaci.2017.05.020 28601681

[9] Bork K Zibat A Ferrari DM Wollnik B Schön MP Wulff K Hereditary angioedema in a single family with specific mutations in both plasminogen and SERPING1 genes. J Dtsch Dermatol Ges. (2020) 18(3):215–23. 10.1111/ddg.14036 32065705

[10] Bork K Wulff K Steinmüller-Magin L Braenne I Staubach-Renz P Witzke G Hereditary angioedema with a mutation in the plasminogen gene. Allergy. (2018) 73(2):442–50. 10.1111/all.13270 28795768

[11] Bork K Wulff K Rossmann H Steinmüller-Magin L Braenne I Witzke G Hereditary angioedema cosegregating with a novel kininogen 1 gene mutation changing the N-terminal cleavage site of bradykinin. Allergy. (2019) 74(12):2479–81. 10.1111/all.13869 31087670

[12] Ariano A D'Apolito M Bova M Bellanti F Loffredo S D'Andrea G A myoferlin gain-of-function variant associates with a new type of hereditary angioedema. Allergy. (2020) 75(11):2989–92. 10.1111/all 32542751

[13] Bork K Wulff K Möhl BS Steinmüller-Magin L Witzke G Hardt J Novel hereditary angioedema linked with a heparan sulfate 3-O-sulfotransferase 6 gene mutation. J Allergy Clin Immunol. (2021) 148(4):1041–8. 10.1016/j.jaci.2021.01.011 33508266

[14] Vincent D Parsopoulou F Martin L Gaboriaud C Demongeot J Loules G Hereditary angioedema with normal C1 inhibitor associated with carboxypeptidase N deficiency. J Allergy Clin Immunol Glob. (2024) 3(2):100223. 10.1016/j.jacig.2024.100223 38445235 PMC10912455

[15] D'Apolito M Santacroce R Vazquez DO Cordisco G Fantini CA D'Andrea G DAB2IP associates with hereditary angioedema: insights into the role of VEGF signaling in HAE pathophysiology. J Allergy Clin Immunol. (2024) 154(3):698–706. 10.1016/j.jaci.2024.05.017 38823490

[16] Ponard D Gaboriaud C Charignon D Ghannam A Wagenaar-Bos IGA Roem D SERPING1 mutation update: mutation spectrum and C1 inhibitor phenotypes. Hum Mutat. (2020) 41(1):38–57. 10.1002/humu.23917 31517426

[17] Wang X Lei S Xu Y Liu S Zhi Y. Mutation update of SERPING1 related to hereditary angioedema in the Chinese population. Hereditas. (2022) 159(1):28. 10.1186/s41065-022-00242-z 35821062 PMC9277798

[18] Liu S Xu Q Xu Y Wang X Zhi Y. Current status of the management of hereditary angioedema in China: a patient-based, cross-sectional survey. Eur J Dermatol. (2020) 30(2):169–76. 10.1684/ejd.2020.3758 32538357

[19] Xu YY Jiang Y Zhi YX Yin J Wang LL Wen LP Clinical features of hereditary angioedema in Chinese patients: new findings and differences from other populations. Eur J Dermatol. (2013) 23(4):500–4. 10.1684/ejd.2013.2105 24001409

[20] Zhou M Luo X Zhou Q Zhou W Zheng R Zhang Y Diagnosis and treatment procedures and health management for patients with hereditary angioedema. Chin J Prev Med. (2023) 57(8):1280–5. 10.3760/cma.j.cn112150-20230509-00359 37574324

[21] Grombirikova H Bily V Soucek P Kramarek M Hakl R Ballonova L Systematic approach revealed SERPING1 splicing-affecting variants to be highly represented in the Czech national HAE cohort. J Clin Immunol. (2023) 43(8):1974–91. 10.1007/s10875-023-01565-w 37620742 PMC10661775

[22] Vatsiou S Zamanakou M Loules G Psarros F Parsopoulou F Csuka D A novel deep intronic SERPING1 variant as a cause of hereditary angioedema due to C1-inhibitor deficiency. Allergol Int. (2020) 69(3):443–9. 10.1016/j.alit.2019.12.009 31959500

[23] Zhi Y An L Lai H Liu R Sun Y Wen L Expert consensus on the diagnosis and treatment of hereditary angioedema. Chin J Allergy Clin Immunol. (2019) 13(1):1–4. 10.3969/j.issn.1673-8705.2019.01.001

[24] Richards S Aziz N Bale S Bick D Das S Gastier-Foster J Standards and guidelines for the interpretation of sequence variants: a joint consensus recommendation of the American college of medical genetics and genomics and the association for molecular pathology. Genet Med. (2015) 17(5):405–24. 10.1038/gim.2015.30 25741868 PMC4544753

[25] Ozkars MY Keskın O Bayram N Onay H Keskın M Bayram H A hereditary angioedema screening in two villages, based on an index case, and identification of a novel mutation, “1033G>T”, at the SERPING1 gene. Postepy Dermatol Alergol. (2019) 36(4):403–11. 10.5114/ada.2018.78898 31616213 PMC6791162

[26] Roche O Blanch A Duponchel C Fontán G Tosi M López-Trascasa M. Hereditary angioedema: the mutation spectrum of SERPING1/C1NH in a large Spanish cohort. Hum Mutat. (2005) 26(2):135–44. 10.1002/humu.20197 15971231

[27] Pedrosa M Phillips-Angles E López-Lera A López-Trascasa M Caballero T. Complement study versus CINH gene testing for the diagnosis of type I hereditary angioedema in children. J Clin Immunol. (2016) 36(1):16–8. 10.1007/s10875-015-0222-9 26661330

[28] Cagini N Veronez CL Constantino-Silva RN Buzolin M Martin RP Grumach AS New mutations in SERPING1 gene of Brazilian patients with hereditary angioedema. Biol Chem. (2016) 397(4):337–44. 10.1515/hsz-2015-0222 26812872

[29] Rijavec M Korošec P Šilar M Zidarn M Miljković J Košnik M. Hereditary angioedema nationwide study in Slovenia reveals four novel mutations in SERPING1 gene. PLoS One. (2013) 8(2):e56712. 10.1371/journal.pone.0056712 23437219 PMC3577750

[30] Andrejević S Korošec P Šilar M Košnik M Mijanović R Bonači-Nikolić B Hereditary angioedema due to C1 inhibitor deficiency in Serbia: two novel mutations and evidence of genotype-phenotype association. PLoS One. (2015) 10(11):e0142174. 10.1371/journal.pone.0142174 26535898 PMC4633032

[31] Rodríguez JA Narváez CF. First analysis of SERPING1 gene in patients with hereditary angioedema in Colombia reveals two genotypic variants in a highly symptomatic individual. J Clin Immunol. (2018) 38(3):294–9. 10.1007/s10875-018-0491-1 29623547

[32] Lumry WR Settipane RA. Hereditary angioedema: epidemiology and burden of disease. Allergy Asthma Proc. (2020) 41(Suppl 1):S08–13. 10.2500/aap.2020.41.200050 33109318

[33] Minafra FG Cunha LAO Mariano RGS Goebel GA de Lima LS Pinto JA. Investigation of mortality of hereditary angioedema in a reference center in Brazil. J Allergy Clin Immunol Pract. (2022) 10(7):1805–12. 10.1016/j.jaip.2022.04.030 35526778

[34] Cao Y Liu S Zhi Y. The natural course of hereditary angioedema in a Chinese cohort. Orphanet J Rare Dis. (2020) 15(1):257. 10.1186/s13023-020-01526-1 32962702 PMC7510061

[35] Liu S Xu Y Liu Y Zhi Y. Hereditary angioedema: a Chinese perspective. Eur J Dermatol. (2019) 29(1):14–20. 10.1684/ejd.2018.3487 30827947

[36] Jia W Suo LM Fan P Dong T Li YJ Ji JM Clinical and genetic studies of a family with hereditary angioedema. Chin J Otorhinolaryngol Head Neck Surg. (2022) 57(8):980–5. 10.3760/cma.j.cn115330-20211209-00789 36058666

[37] Qu L Fu Y Sha S Zou Q Liu M Xiao T Mutation analysis of C1 inhibitor (C1INH) gene in a Chinese family with hereditary angioedema. Chin J Derm Venereol. (2019) 33(8):881–4. 10.13735/j.cjdv.1001-7089.201812089

[38] Du W Yang K Zhang Q Lin X Zhang W Guo W Case report: identification of a novel mutation, c.1067T>A, in the SERPING1 gene in a Chinese male with type 1 hereditary angioedema. Front Allergy. (2025) 6:1554940. 10.3389/falgy.2025.1554940 40364801 PMC12069465

[39] Zhang W Liu H. Identification of a novel mutation in the SERPING1 gene in a 17-year-old Chinese girl with type I hereditary angioedema. JAAD Case Rep. (2024) 56:38–9. 10.1016/j.jdcr.2024.10.023 39839458 PMC11750429

[40] Du W Yang K Li C Zhang Q Lin X Liu H Variation analysis of C1 inhibitor gene in a family with hereditary angioedema. Chin J Derm Venereol. (2024) 38(12):1327–32. 10.13735/j.cjdv.1001-7089.202403067

